# *Candida* Empyema as a Red Flag for Esophageal Rupture: A Case Report and Literature Review

**DOI:** 10.1155/2020/3935691

**Published:** 2020-04-14

**Authors:** Naureen Ali, Getahun Abate

**Affiliations:** Division of Infectious Diseases, Allergy and Immunology, Saint Louis University, St. Louis, USA

## Abstract

*Background*. *Candida* empyema is a rare entity with an extremely high mortality rate.We present a case of multi species *Candida* empyema in an immunocompetent female patient with Boerhaave syndrome secondary to retching and vomiting after heroin withdrawal. This case highlights an unusual presentation of a common infection but stresses on the fact that a high index of suspicion is necessary for early identification, prompt initiation of antifungal therapy, and drainage with surgical repair to improve overall survival and outcomes.

## 1. Background

*Candida* empyema is a rare entity with a crude mortality rate as high as 62% [1]. Patients with underlying malignancies such as esophageal, gastric, and breast cancer are more susceptible to this condition. Other risk factors include recent contiguous surgery including lung and heart transplants [2]. Our patient was a 30-year-old immunocompetent female who developed *Candida* empyema secondary to Boerhaave syndrome.

## 2. Case Report

Our patient was a 30-year-old Caucasian female with a past medical history of bipolar disorder, depression, generalized anxiety disorder, intravenous heroin use, and migraines who presented to an outside hospital emergency department with acute onset shortness of breath and vomiting. Her symptoms started 2 days prior to presentation after she abruptly stopped using heroin in an attempt to quit. The patient had an extensive history of intravenous drug use. She started using intravenous drugs at 14 years of age, initially using only benzodiazepines and then intravenous heroin. She injected heroin in the large veins of her arms, legs, and neck. She however denied sharing needles. She had been in rehabilitation facilities multiple times in the past and had received methadone for her heroin dependence intermittently during the 4-5 years prior to presentation.

The patient has been having vomiting more than 5 times a day with associated retching. The vomitus was bilious. She was also having shortness of breath which was worsening. In the emergency department, she was afebrile with oral temperature of 98°F but was found to be tachypneic with respiratory rate of 24 breaths per minute and hypoxic with oxygen saturation of 87% on 15 L of oxygen via non-rebreather mask. She was subsequently placed on Bilevel Positive Airway Pressure (BiPAP) for her respiratory distress. Physical examination was only remarkable for bilateral diffuse wheezing and rhonchi.

Initial laboratory workup showed a lactic acid level of 3.05 mmol/L and white blood cell count of 23.0 × 10^3^/*μ*L. A urine drug screen was positive for opiates. Given the concern for sepsis and aspiration pneumonia, blood cultures were obtained and patient was empirically started on intravenous vancomycin (with a goal trough of 15–20 mg/L) and extended infusion piperacillin tazobactam (3.375 mg every 6 hours). Her initial chest X-ray was normal. Treatment for opiate withdrawal at this time was also initiated. After admission to the hospital, she developed a new severe upper back pain and continued to require BiPAP.

A repeat chest X-ray performed on day 2 of her admission at the outside hospital showed a 2.7 cm right-sided pneumothorax (measured from the anterior chest wall) and bilateral small pleural effusions. Another chest X-ray about 3 hours later showed that the pneumothorax was persistent, and therefore a right-sided chest tube was placed with approximately 700 mL of fluid removed. Pleural fluid was cloudy in color, with WBC count of 20,200 × 10E6/L (94% Neutrophils), pH 6.00, LDH 837 U/L, glucose 28 mg/dL, and protein 2.5 g/dL. Serum protein resulted as 9.7 g/dL. The findings were consistent with an exudative effusion or empyema.

On day 3, she developed dysphagia and had increased oral secretions, and had to be intubated for air way protection. Postintubation chest X-ray showed increased width of right-sided pneumothorax to 6.65 cm and a kinked chest tube. The chest tube was manipulated, and it drained another 1.0 L of pleural fluid. Overnight, the patient's oxygen requirement increased, necessitating FiO_2_ change to 90% and positive end expiratory pressure (PEEP) of 10 cmH_2_O. The patient was subsequently transferred to a tertiary care hospital for further management.

At our hospital, a repeat chest X-ray was obtained which showed that the chest tube and endotracheal tube were in place. There was now a left-sided pleural effusion, but the right-sided pneumothorax and effusion had apparently resolved. Antibiotics were switched to ampicillin-sulbactam (3 g intravenous every 6 hours) for suspected aspiration pneumonia. Other laboratory tests indicated that patient was negative for human immunodeficiency virus and positive for hepatitis C.

**Figure 1:**
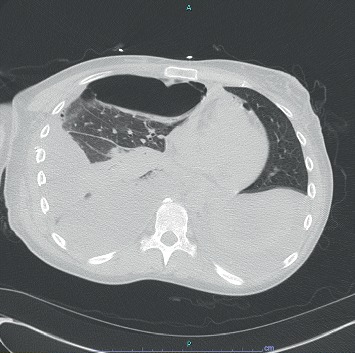
Computed tomography (CT) of the chest without contrast: moderate-large loculated right pneumothorax anteriorly with mediastinal shift to the left suggesting tension component and small volume pneumomediastinum.

**Figure 2:**
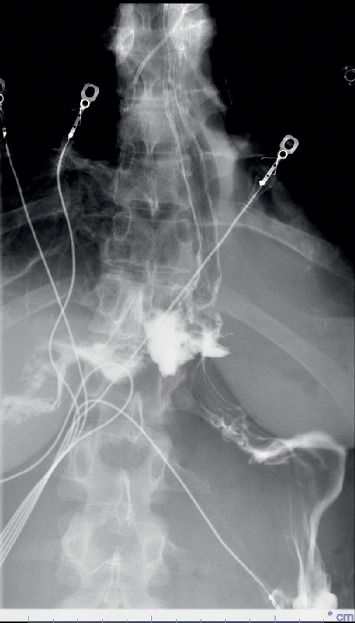
Esophagogram: esophageal perforation with contrast extravasation at the approximate level of the gastroesophageal junction.

On day 2 of admission to our hospital (i.e., day 4 since presentation), the patient's condition improved. She was extubated and oxygen nasal cannula was placed. Pleural fluid obtained at the outside hospital grew *Candida albicans*, and therefore intravenous micafungin (100 mg daily) was initiated. At this point, the Infectious Diseases team was consulted for possible *Candida* empyema. It was found that patient's main initial problem soon after she stopped using heroin was severe retching. She was unable to sleep because of retching. She subsequently developed worsening shortness of breath. On examination, she was noted to have a whitish coating on the tongue and oral mucosa suggestive of oral thrush. A CT of the chest was obtained which showed a right apical chest tube within a large loculated appearing right pleural effusion containing locules of gas, likely representing empyema and a moderate to large loculated right pneumothorax anteriorly with mediastinal shift to the left (Figure 1). The patient was having fever with a maximum oral temperature of 102.7°F. The chest tube remained in place during this time. Antibiotics were changed to intravenous meropenem 1 gram every 8 hours and intravenous fluconazole (400 mg daily). Because of history of sever retching, oral thrush, pneumothorax and *Candida* empyema, esophageal perforation was suspected. An esophagogram with water soluble contrast was obtained and confirmed esophageal perforation with contrast extravasation at the level of gastroesophageal junction (Figure 2).

On day 4 at our hospital (i.e., day 6 since presentation), the patient underwent a right thoracotomy with decortication and repair of esophageal perforation about 3–5 centimeters above the hiatus. Gross pus and gelatinous material were found in the pleural cavity which were evacuated and sent for culture.

On day 6 at our hospital (i.e., day 8 since presentation), the results of pleural fluid culture obtained at the outside hospital were updated and reported to grow *Candida albicans, Candida dubliniensis, Candida glabrata, and Bacillus* species (not cereus or anthracis). *Candida albicans* was susceptible to anidulafungin, caspofungin, fluconazole with minimum inhibitory concentration (MIC) of 0.25 *μ*g/mL, micafungin, and voriconazole. There were no established Clinical and Laboratory Standards Institute (CLSI) breakpoints to interpret susceptibility of *Candida dubliniensis,* but MICs for the above drugs were <0.08–0.5 which were in susceptible range if CLSI standards for *Candida albicans* were used. *Candida glabrata* was susceptible to anidulafungin, caspofungin, fluconazole (MIC 16), and micafungin. *Bacillus* species was sensitive to meropenem, levofloxacin, trimethoprim sulfamethoxazole, vancomycin, and gentamycin and resistant to penicillin and clindamycin.

Surgical samples obtained during the decortication grew *Candida albicans, Candida dubliniensis*, and *Streptococcus viridans* group (sensitivity testing was not done). Multiple routine blood and blood fungal cultures obtained throughout the admission remained negative. A transthoracic echocardiogram (TTE) was performed and was negative for cardiac valvular vegetations. Therefore, because of negative blood cultures and a negative TTE, endocarditis was considered less likely. A transesophageal echocardiogram could not be performed because of esophageal perforation.

In the following days, the patient's condition improved, and she was switched to intravenous vancomycin (with a goal trough of 15–20 *μ*g/mL), intravenous ampicillin-sulbactam (3 g every 6 hours) and intravenous micafungin (100 mg daily) to complete 3 weeks of therapy from initiation of appropriate antifungal agent (a total of 4 weeks of therapy). A repeat chest CT scan prior to completion of antibiotic therapy demonstrated pleural-based scarring in the lung bases on the right with minimal atelectatic changes. There was no evidence of pneumomediastinum, pneumothorax, or recurrent rupture of the esophagus.

## 3. Discussion

Here we present a case of *Candida* empyema in a patient with history of severe retching, oral thrush, and esophageal rupture. Our patient was an otherwise healthy female who developed persistent vomiting and retching while attempting to quit heroin use at home. *Candida* empyema is extremely rare and has been reported to occur due to inoculation of the pleural space by *Candida* species secondary to esophageal perforation, extension of abdominal or pulmonary infections, thoracic or abdominal surgical interventions, or from repeated thoracentesis procedures. [1].

In our case, we became concerned about possible esophageal rupture due to history of severe retching, worsening shortness of breath, persistent inability to swallow, oral thrush, and *Candida* empyema. Given the significant oral thrush on examination and pleural fluid cultures also growing *Candida,* our suspicion for a possible perforation was high which prompted us to pursue an esophagogram, resulting in the eventual diagnosis.

Culture results of *Candida* typically take several days, particularly to report sensitivities. Our patient had multiple *Candida* species isolated from her pleural fluid including *Candida albicans, Candida dubliniensis,* and *Candida glabrata* which is extremely rare [3]. Only a handful of cases have been reported in literature with esophageal or gastric ulcer perforation as the cause of *Candida* empyema [4].

Patients with fungal empyema generally benefit from surgery and systemic antifungal therapy. There is an increased risk of morbidity and mortality in immunocompromised patients particularly those with respiratory failure [5], hence early identification, initiation of antifungal therapy, drainage, and surgical repair is necessary to improve overall morbidity and mortality. Multiple studies have identified *Candida albicans* as the most common isolate [1, 6]. Because *Candida* empyema is rare, there are no randomized controlled trials to guide medical or surgical management of these cases. There are, however, case series and single center experience reports which indicate that 2–4 weeks of antibiotic treatment are usually used [1, 2]. Despite appropriate medical treatment, the mortality can be as high as 54% [1, 2] indicating the need for more aggressive work up and management. Physicians should have a high index of suspicion for esophageal perforation in patients presenting with pneumothorax in a setting of recent severe retching. Esophago or gastropleural fistulas should be considered as possible causes of *Candida* empyema. In some cases, patients may have bacterial superinfection of the empyema as seen here with *Bacillus* and *Streptococcus viridans* group isolated from patient's pleural fluid.

## 4. Conclusion

In summary, our case demonstrates that *Candida* empyema is a rare but serious complication of Boerhaave syndrome. High risk and immunocompromised patients have a greater predisposition to fungal infections and are more susceptible to this entity.
